# Supplementary material for: Porphyromonas gingivalis HmuY and Streptococcus gordonii GAPDH—Novel Heme Acquisition Strategy in the Oral Microbiome
Source: Int J Mol Sci. 2020 Jun 10;21(11):4150. doi: 10.3390/ijms21114150 (PMC7312356; doi:10.3390/ijms21114150)
Supplement: Supplementary file 1 [file ijms-21-04150-s001.zip › SgGAPDH Supplementary table IJMS final.pdf]

Supplementary materials

Porphyromonas gingivalis HmuY and *Streptococcus gordonii* GAPDH – novel heme acquisition strategy in the oral microbiome

Paulina Ślęzak [1,&], Michał Śmiga [1,&], John W. Smalley [2] , Klaudia Siemińska [1] and Teresa Olczak [1,*]

[1] Laboratory of Medical Biology, Faculty of Biotechnology, University of Wrocław, 14A F. Joliot-Curie St., 50-383 Wrocław, Poland; teresa.olczak@uwr.edu.pl

[2] School of Dentistry, Institute of Clinical Sciences, University of Liverpool, Daulby St., Liverpool L69 3GN, U.K.; josmall@liv.ac.uk

Table S1. List of primers used in this study

Name	5′→3′ DNA sequence	Description (reference)
F_HiFi_pTXB1	CTTTAAGAAGGAGATATACATATGGTAGTTAAAGTTGGTATTAAC	Primers used to amplify the gene encoding SgGAPDH with C-terminal intein and chitin-binding domain (this study)
R_HiFi_pTXB1	ACTAGTGCATCTCCCGTGATGCATTTAGCGATTTTCGCG	
F_SgGAPDH_pTriEx	ACACCATCACCACCATCACTTAATCGAGGGAAGGATGGTAGTTAAAGTTGGTATTAAC	Primers used to amplify the gene encoding SgGAPDH with N-terminal His-tag and Factor X recognition site (this study)
R_SgGAPDH_pTriEx	GAGATCTGAGAATTCGGATCCTTATTTAGCGATTTTCGCGAAG	
F_pMAL-Hc5x_Tfo	CAACCTCGGGATCGAGGGAAGGATGGACAAGAAAGACGACGT	Primers used to amplify the gene encoding Tfo protein with N-terminal 6His-MBP (this study)
R_pMAL-Hc5x_Tfo	CTTATTTAATTACCTGCAGGGAATTCGGATCCTTATTTCGGTTGAAATTCGTAATTAAAAGA	
F_H43_GAPDH	CCTTACAGATCCTGTAATGCTTGCAGCTCTGTTGAAATATGACAC	Site-directed mutagenesis primers used to introduce His43Ala substitution (this study)
R_H43_GAPDH	GTGTCATATTTCAACAGAGCTGCAAGCATTACAGGATCTGTAAGG	
F_H109_GAPDH	AAAGCAGCTGCTGAAAAAGCGTTGCATGCTGGTGGTGCT	Site-directed mutagenesis primers used to introduce His109Ala substitution (this study)
R_H109_GAPDH	AGCACCACCAGCATGCAACGCTTTTTCAGCAGCTGCTTT	
F_H111_GAPDH	GCTGCTGAAAAACACTTGGCTGCTGGTGGTGCTAAGAAAGTTG	Site-directed mutagenesis primers used to introduce His111Ala substitution (this study)
R_H111_GAPDH	CAACTTTCTTAGCACCACCAGCAGCCAAGTGTTTTTCAGCAGC	

F_H137_GAPDH	AAACAGTCGTATTTAACACTAACGCGGATATTCTTGATGGTACTGAAA	Site-directed mutagenesis primers used to introduce His137Ala substitution (this study)
R_H137_GAPDH	TTTCAGTACCATCAAGAATATCCGCGTTAGTGTTAAATACGACTGTTT	
F_H179_GAPDH	GAAGGATTGATGACTACTATCGCGGCTTACACTGGTGACCAA	Site-directed mutagenesis primers used to introduce His179Ala substitution (this study)
R_H179_GAPDH	TTGGTCACCAGTGTAAGCCGCGATAGTAGTCATCAATCCTTC	
F_H192_GAPDH	GATCCTTGACGGTCCAGCCCGTAAAGGTGACCTTCGTC	Site-directed mutagenesis primers used to introduce His192Ala substitution (this study)
R_H192_GAPDH	GACGAAGGTCACCTTTACGGGCTGGACCGTCAAGGATC	
HYq4-F	GCTTCGAAATACGAAACGTG	Primers used in RT-qPCR experiments to amplify fragment of the *hmuY* gene [79]
HYq4-R	TATATCCGTCTGTCGGAACG	
F_hmuR	CTACCGACACCATCGTATCC	Primers used in RT-qPCR experiments to amplify fragment of the *hmuR* gene [72]
R_hmuR	CATTGAGCTGATCTCTGGAAC	
16SrRNA–F	CTTGACTTCAGTGGCGGCAG	Primers used in RT-qPCR experiments to amplify fragment of the *16SrRNA* gene [80]
16SrRNA–R	AGGGAAGACGGTTTTCACCA	

72. Smiga, M., Stepien, P., Olczak, M. & Olczak, T. PgFur participates differentially in expression of virulence factors in more virulent A7436 and less virulent ATCC 33277 *Porphyromonas gingivalis* strains. *BMC Microbiol*. **19**:127 (2019).
79. Gmiterek, A. et al. The unique *hmuY* gene sequence as a specific marker of *Porphyromonas gingivalis* infection. *PLoS One* **8(7)**:e67719 (2013).
80. Maeda, H. et al. Quantitative real-time PCR using TaqMan and SYBR Green for *Actinobacillus actinomycetemcomitans*, *Porphyromonas gingivalis*, *Prevotella intermedia*, *tetQ* gene and total bacteria. *FEMS Immunol. Med. Microbiol.* **39**, 81-86 (2003).
