# Supplementary material for: Porphyromonas gingivalis HmuY and Streptococcus gordonii GAPDH—Novel Heme Acquisition Strategy in the Oral Microbiome
Source: Int J Mol Sci. 2020 Jun 10;21(11):4150. doi: 10.3390/ijms21114150 (PMC7312356; doi:10.3390/ijms21114150)
Supplement: Supplementary file 1 [file ijms-21-04150-s001.zip › SgGAPDH and HmuY Supplementary figures IJMS.pdf]

Supplementary materials

Porphyromonas gingivalis HmuY and *Streptococcus gordonii* GAPDH – novel heme acquisition strategy in the oral microbiome

Paulina Ślęzak [1,&], Michał Śmiga [1,&], John W. Smalley [2] , Klaudia Siemińska [1] and Teresa Olczak [1,*]

[1] Laboratory of Medical Biology, Faculty of Biotechnology, University of Wrocław, 14A F. Joliot-Curie St., 50-383 Wrocław, Poland; teresa.olczak@uwr.edu.pl

[2] School of Dentistry, Institute of Clinical Sciences, University of Liverpool, Daulby St., Liverpool L69 3GN, U.K.; josmall@liv.ac.uk

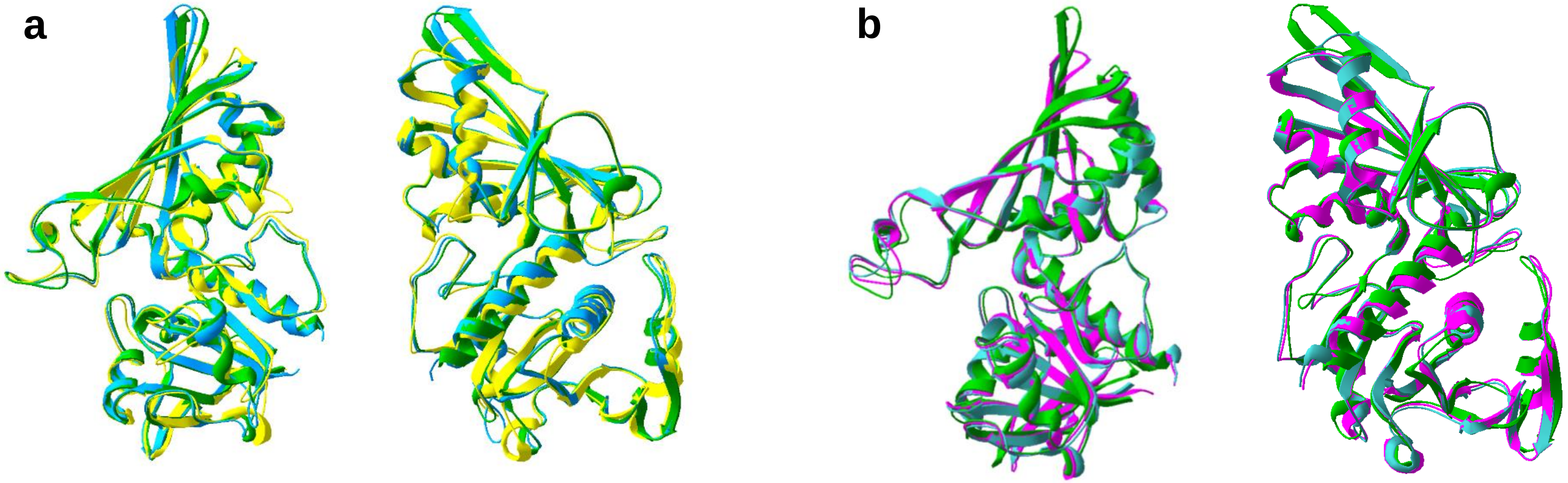

Figure S1. Theoretical three-dimensional model of SgGAPDH protein structure. (**a**) Comparison of SgGAPDH theoretical model (green) with the protein structures of GAPDH from *Streptococcus agalactiae* (PDB: 4QX6, blue, 92% homology with SgGAPDH) and GAPDH from *Borrelia burgdorferi* (PDB: 5LD5, yellow, 51% homology with SgGAPDH) is shown. (**b**) Comparison of SgGAPDH theoretical model (green) with the protein structures of human GAPDH (PDB: 1ZNQ, pink, 46% homology with SgGAPDH) and rabbit GAPDH (PDB: 1J0X, cyan, 47% homology with SgGAPDH).

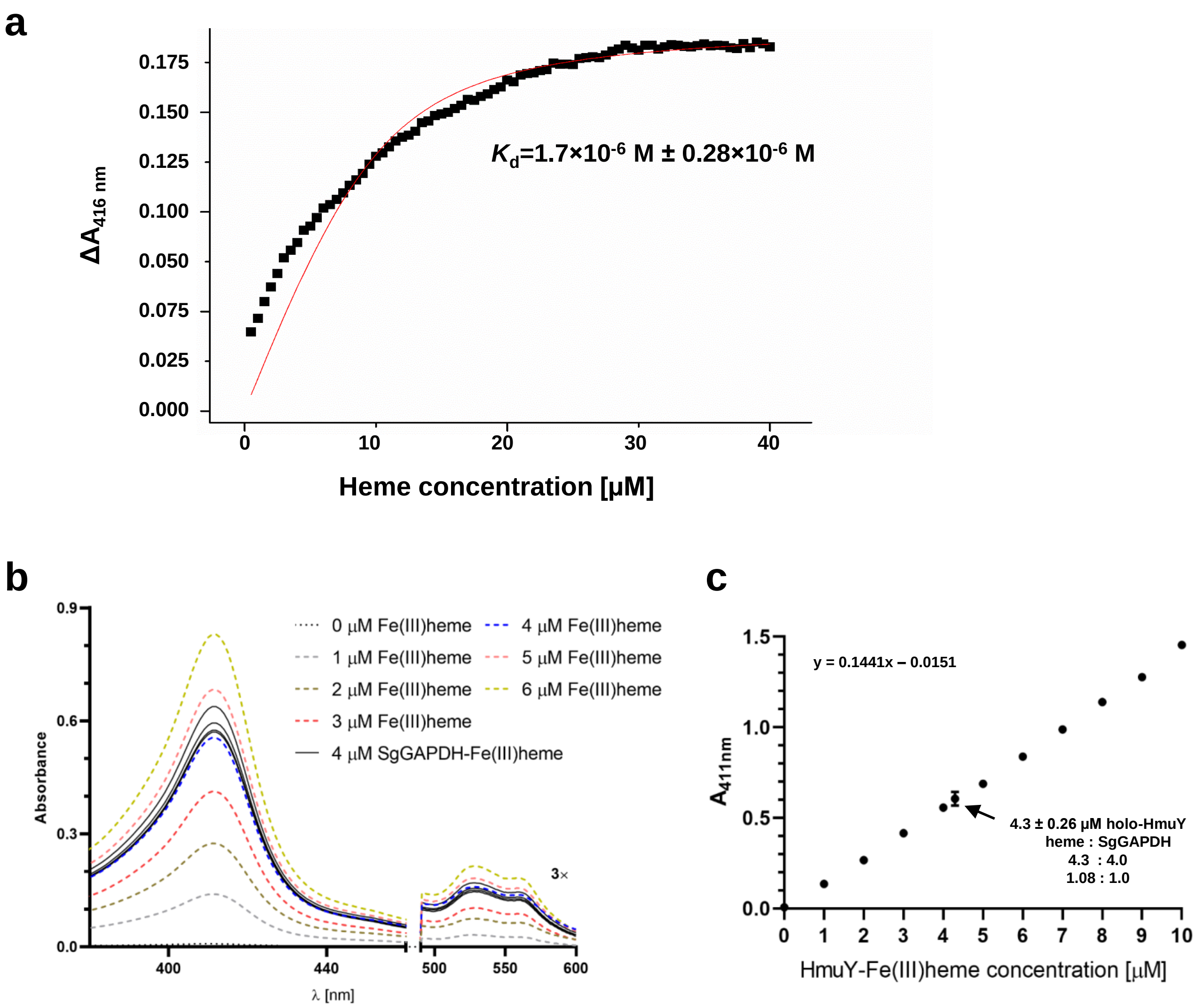

Figure S2. Determination of the heme binding ratio to SgGAPDH. (**a**) Titration of SgGAPDH protein with heme was carried out to determine the dissociation constant (K_d). 10 µM SgGAPDH was titrated with Fe(III)heme and the difference spectra at 416 nm between samples of protein-heme complex and heme alone were used to construct the titration curve. Results are shown as mean ± SD from three independent experiments. (**b**, **c**) To confirm protein:heme ratio, determination of the amount of bound heme per SgGAPDH monomer was performed using alternative method. 4 µM SgGAPDH-Fe(III)heme complex was mixed with 25 µM apo-HmuY and incubated for 30 minutes. UV-visible spectrum was examined and shown as black, solid line in (**b**). Dashed lines show titration of mixed 25 µM apo-HmuY and 4 µM apo-SgGAPDH with Fe(III)heme. The absorbance at 411 nm, corresponding to the HmuY-heme complex maximum in the Soret region, was used to create a standard curve of the amount of heme bound to the HmuY protein shown in (**c**).

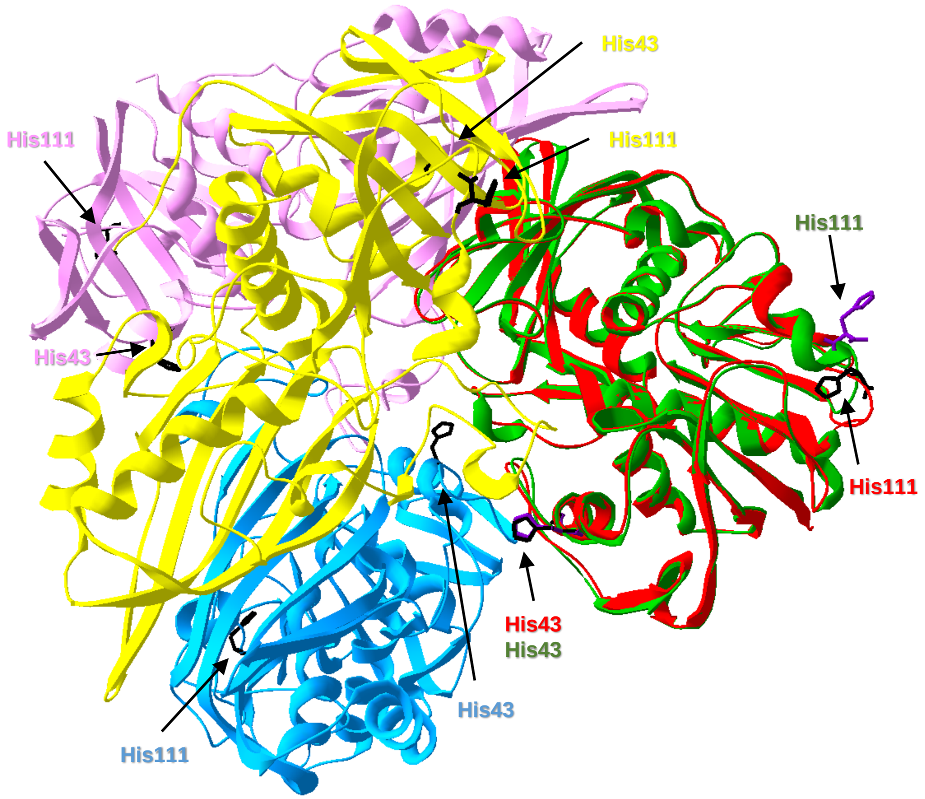

Figure S3. Theoretical SgGAPDH model (green) overlaid with the structure of GAPDH protein tetramer from *Streptococcus agalactiae* (PDB: 4QX6). Monomers of *S. agalactiae* GAPDH are shown in different colors (red, yellow, pink and blue). Potential heme-binding His residues are drawn in black for *S. agalactiae* GAPDH and in violet for SgGAPDH.

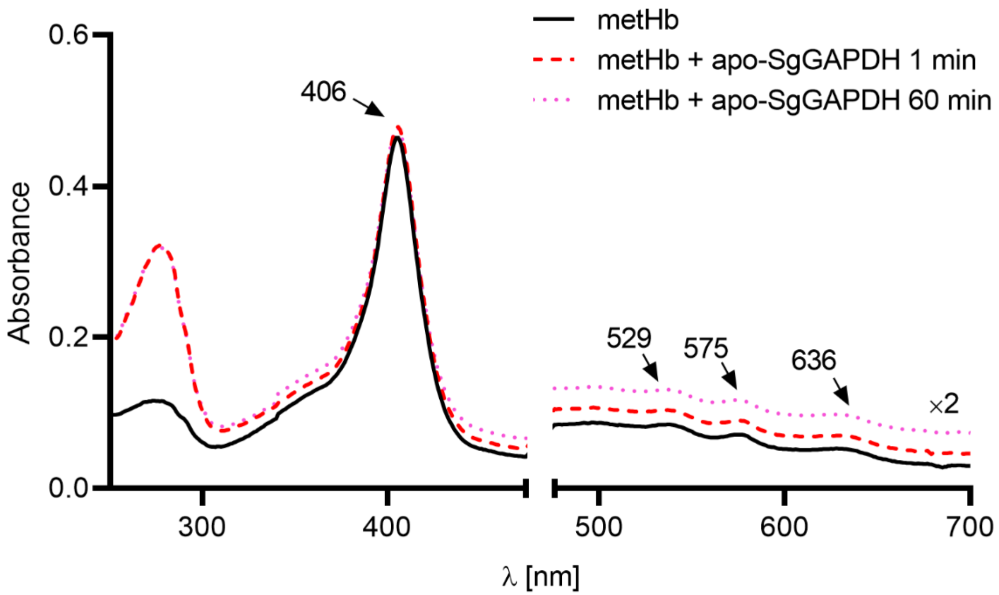

Figure S4. Heme sequestration from methemoglobin by SgGAPDH. 5 µM methemoglobin (metHb) was mixed with 5 µM apo-SgGAPDH protein. Spectra were recorded at indicated time points.

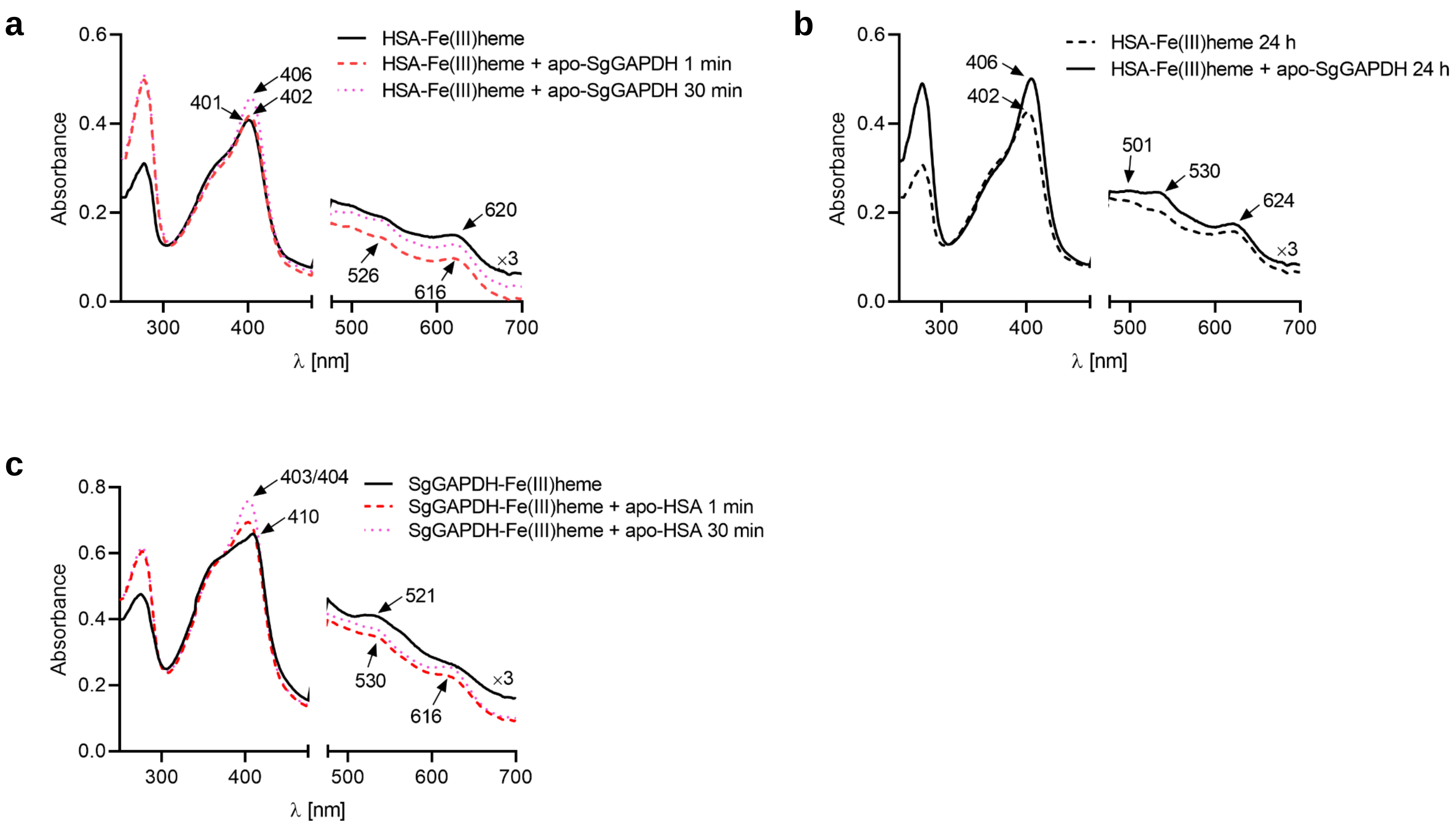

Figure S5. Heme sequestration from human serum albumin-heme complex by SgGAPDH. (**a, b**) 5 µM human serum albumin (HSA) complex with Fe(III)heme was mixed with apo-SgGAPDH protein. (**c**) Alternatively, 5 µM SgGAPDH-Fe(III)heme complex was mixed with apo-HSA protein. Spectra were recorded at indicated time points.

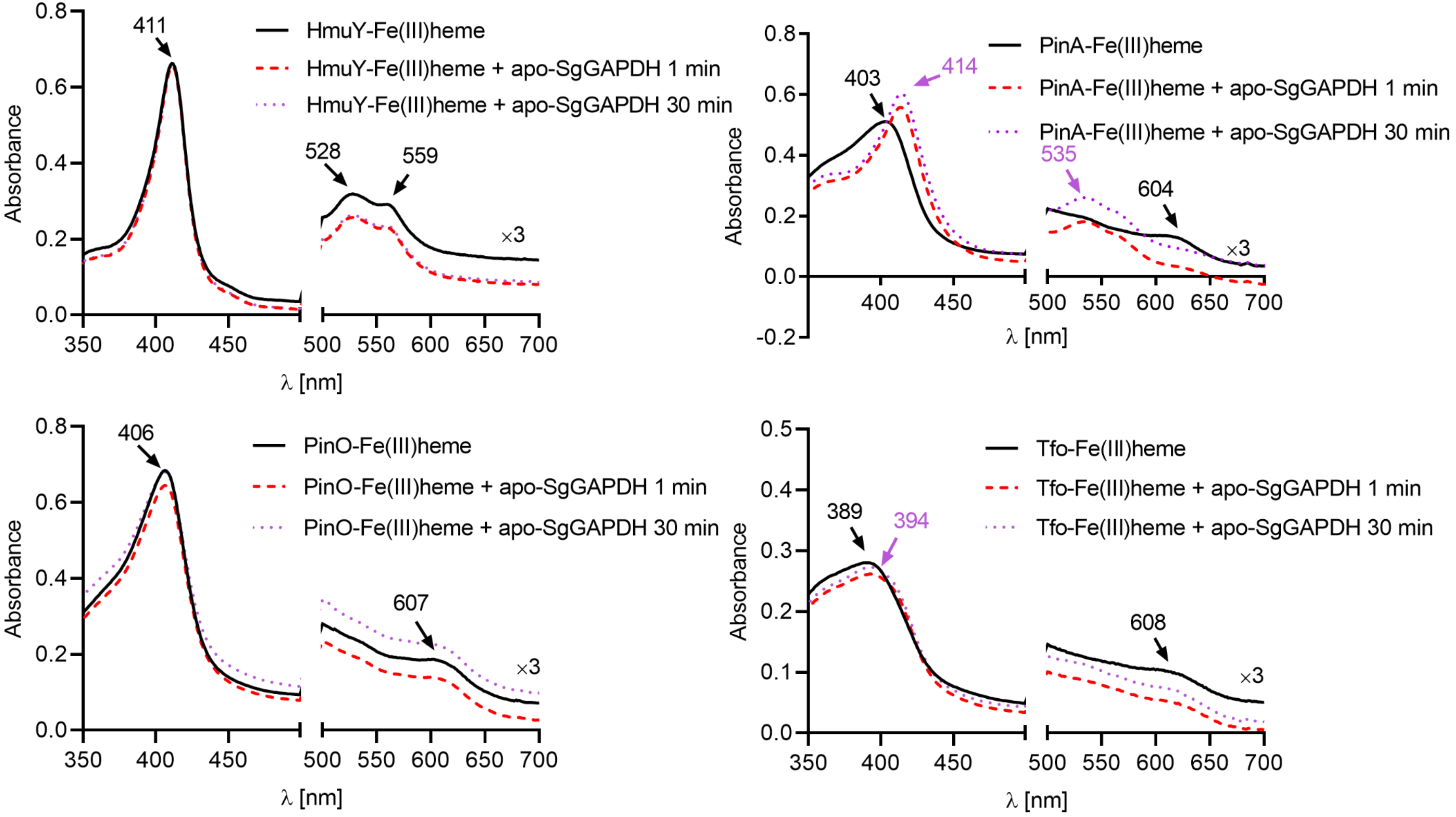

Figure S6. Heme sequestration by SgGAPDH from HmuY and its homologs complexed with heme under air (oxidizing) conditions. Apo-SgGAPDH (10 μM) was incubated with equimolar concentration of HmuY-, PinO-, PinA- or Tfo-heme complexes. Changes in absorption spectra analyzed by UV-visible spectroscopy are shown at indicated time points.

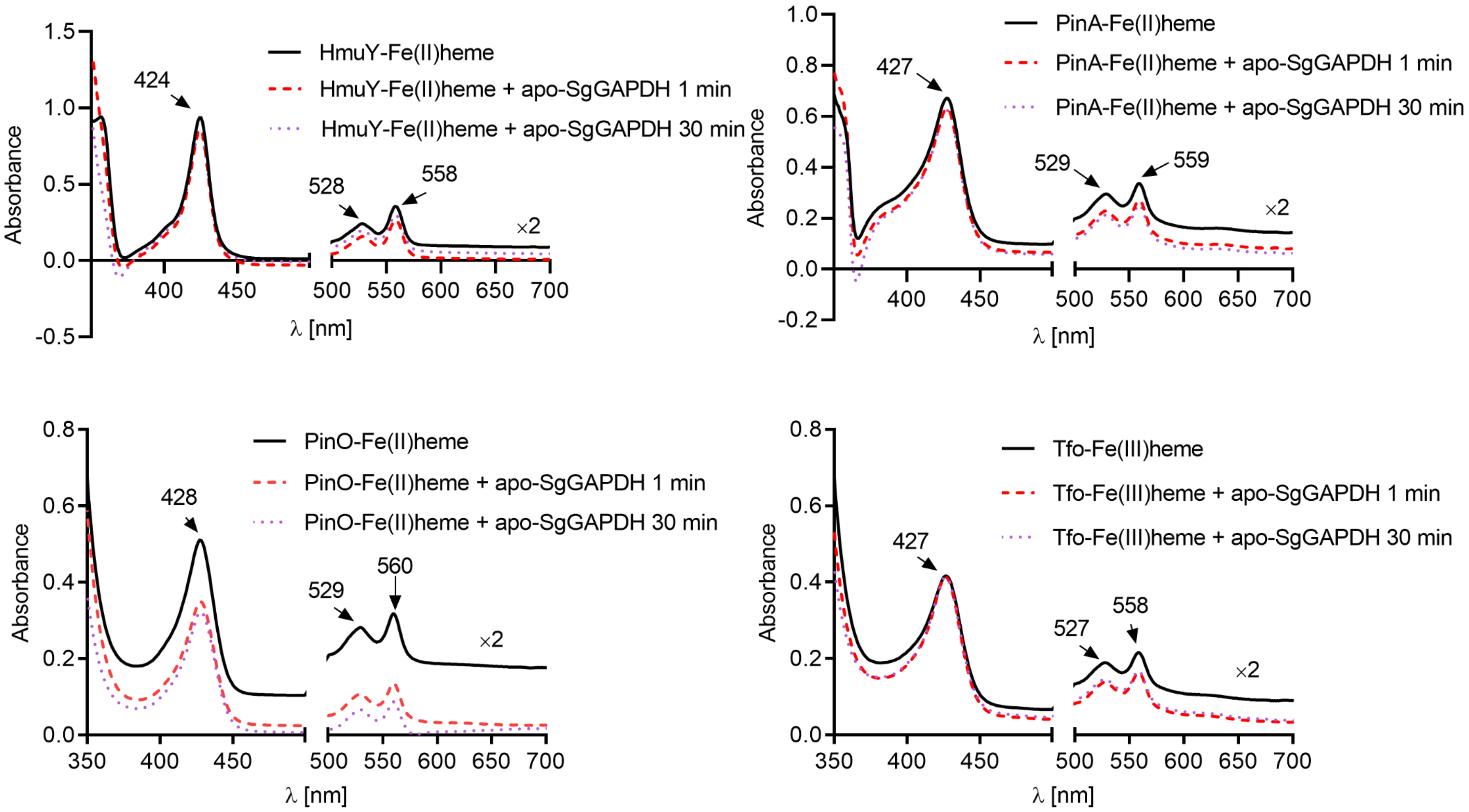

Figure S7. Heme sequestration by SgGAPDH from HmuY and its homologs complexed with heme under reducing conditions. Apo-SgGAPDH (10 μM) was incubated with equimolar concentration of HmuY-, PinO-, PinA- or Tfo-heme complexes. Changes in absorption spectra analyzed by UV-visible spectroscopy are shown at indicated time points.

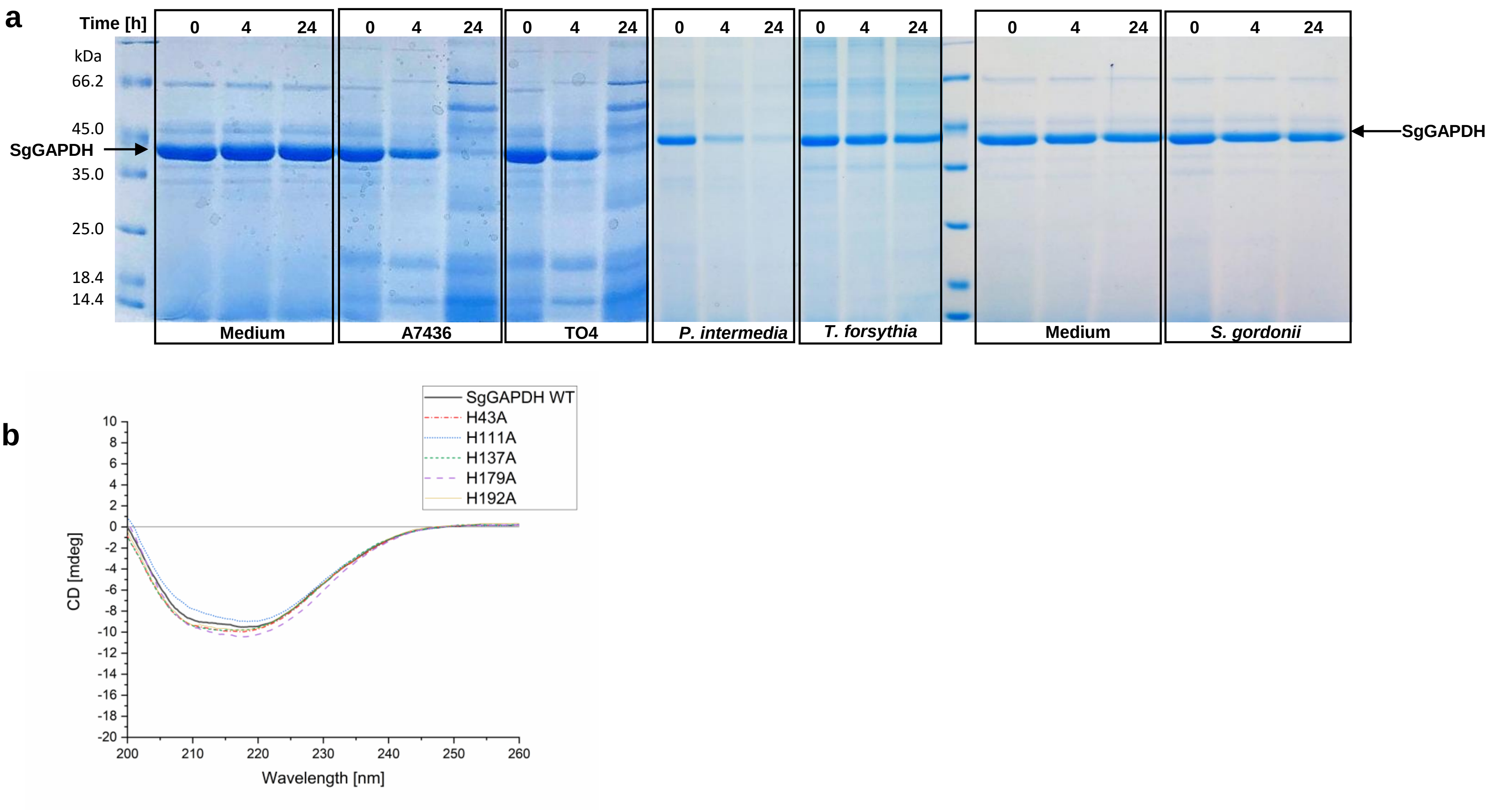

Figure S8. Stability analysis of SgGAPDH. (**a**) Susceptibility of SgGAPDH to proteases produced by bacterial cultures was examined by growing bacteria under high-iron/heme conditions (Hm) in the presence of 5 µM purified SgGAPDH protein (marked with arrows). Samples collected at indicated time points were subjected to SDS-PAGE and proteins were stained with Coomassie Brilliant Blue G-250. A7436, wild type *P. gingivalis* strain; TO4, Δ*hmuY* deletion mutant strain constructed in A7436 strain. (**b**) Analysis of secondary structure of SgGAPDH site-directed mutagenesis protein variants examined by CD spectroscopy.
